# Inter-Relationship between Rhinitis and Conjunctivitis in Allergic Rhinoconjunctivitis and Associated Risk Factors in Rural UK Children

**DOI:** 10.1371/journal.pone.0143651

**Published:** 2015-11-24

**Authors:** Michael R. Perkin, Tara Bader, Alicja R. Rudnicka, David P. Strachan, Christopher G. Owen

**Affiliations:** Population Health Research Institute, St George’s, University of London, Cranmer Terrace, London, SW17 ORE, United Kingdom; Beijing Institiute of Otolaryngology, CHINA

## Abstract

**Objective:**

Allergic conjunctivitis (AC) is a common condition, especially in childhood. The extent to which it occurs concurrently with or independently from allergic rhinitis (AR) has not been well described.

**Aim:**

To examine the inter-relationship between rhinitis and conjunctivitis and the epidemiological risk factors for these conditions in a rural UK population.

**Methods:**

Cross-sectional study of rural school children (aged 5–11 years). Parental questionnaires were used to diagnose allergic outcomes (including conjunctivitis, rhinitis and rhinoconjunctivitis), and to collect data on atopic history, demographic and environmental exposures. Odds ratios of allergic outcome by exposure were examined adjusted for age, sex, breastfeeding, family history of allergy, number of older and younger siblings.

**Results:**

Prevalence of conjunctivitis was 17.5%, rhinitis 15.1% and rhinoconjunctivitis 13.0%. Seasonality of symptoms varied by condition: 64.7% of those with conjunctivitis had seasonal symptoms (April-Sept only), 46.7% of those with rhinitis and 92.2% of those with rhinoconjunctivitis. Living on a farm consistently reduced the risk of conjunctivitis (odds ratio 0.47, 95%CI 0.29–0.79, p = 0.004), rhinitis (OR 0.57, 95%CI 0.33–1.01, p = 0.05) and rhinoconjunctivitis (OR 0.57, 95%CI 0.32–1.03, p = 0.06). Exposure to farm animals (particularly in early life), current consumption of unpasteurised milk and playing in a barn or stable significantly reduced the risk of all three conditions.

**Conclusion:**

More children had parent-reported conjunctivitis than rhinitis. The majority of children with either condition also reported symptoms with the other condition. Farmers’ children have less eye and/or nasal symptoms. A number of farming variables linked with the farm microbial environment are likely to be mediating the protective effect.

## Introduction

Allergic conjunctivitis (AC) accounts for 15% of eye related consultations in primary care [1]. Most will include those with acute forms of the condition, which are either seasonal or perennial [2]. Seasonal allergic conjunctivitis (SAC), a Type 1 IgE mediated hypersensitivity reaction, is commonly seen when pollens are present in the atmosphere (typically during spring and summer months). It is estimated that the population prevalence of SAC is between 16–20%, but most appear to self-manage the condition (often by avoidance of allergens and/or with use of over the counter medications) with only 10–12% of SAC patients seeking medical attention [3]. Hence, while the cost of allergic conditions to the National Health Service is considerable (estimated at £1 billion per year), this is likely to underestimate the spectrum of disease in the population at large [4]. Data are not available for SAC but seasonal allergic rhinitis (SAR) in childhood has been associated with poorer academic performance [5].

The Nomenclature Review Committee of the World Allergy Organization (WAO) in its revised nomenclature for allergy for global use stated that: “Hypersensitivity symptoms from the nose, e.g., itching, sneezing, increased secretion, and blockage, when immunologically mediated, should be called *allergic rhinitis*. Because the great majority of cases are IgE-antibody-mediated, a proper term would be *IgE-mediated allergic rhinitis*”.[6] However “great majority” is not referenced or more explicitly determined. The WAO further states: “If the symptoms are seasonal, e.g., pollen-induced allergic rhinitis, *seasonal allergic rhinitis* is an appropriate term” [6]. It is uncertain the extent to which parents are able to successfully recognise when their children have IgE mediated pollen induced rhinitis and/or conjunctivitis symptoms. Sensitization to pollen can occur in the absence of symptoms and seasonal symptoms with a perceived pollen trigger can occur in the absence of pollen sensitization.

IgE-mediated AC commonly accompanies AR, and the WAO proposed that the disorder be appropriately termed allergic rhinoconjunctivitis (ARC).[6] The extent of overlap between rhinitis and conjunctivitis was not more precisely stated. The report also acknowledged that the relationship between allergic and non-allergic conjunctivitis requires further investigation.[6]

The epidemiology of ocular allergy in an adult population was explored in the National Health And Nutrition Examination Survey III [7]. 6.4% reported ocular symptoms, 16.5% nasal symptoms and 29.7% both. Forty percent reported at least 1 occurrence of ocular symptoms in the last 12 months.

Rhinitis and rhinoconjuctivitis have been extensively studied under the auspices of the International Study of Asthma and Allergies in Children (ISAAC) [8]. However, the prevalence of SAC without rhinitis and rhinoconjunctivitis has not been quantified in large populations of UK children. The original ISAAC questions prohibit investigating this issue as the presence or absence of “itchy-watery eyes” was only asked as a nested question to those who had given an affirmative response to their rhinitis question.

Examining allergic disorders in early life is of interest as this is a period when susceptibility to allergens might be influenced. We aimed to investigate the prevalence of rhinitis, conjunctivitis and the inter-relationship between the two conditions. We explored the extent to which parent suspicions of environmental triggers overlapped with sensitization on skin prick testing. This study took place within a rural population of children. There has been increasing interest in the potentially protective influence of exposure to farming in early life on allergic outcomes [9,10], possibly through modulation of cytokine production [11]. While these associations have been examined for AR and ARC [12,13], associations with SAC have not been specifically examined; this is of particular interest to see if associations observed with other allergic outcomes can be replicated.

## Materials and Methods

### Study Population

This investigation was carried out within the Study of Asthma and Allergy in Shropshire, a school based survey examining whether farming and animal related exposures were associated with allergy. Full details of the cross sectional study design have been reported elsewhere [13]. In brief, the study was based in 73 primary schools (7226 pupils) within the county of Shropshire. Shropshire was chosen for its high density of farming, with 86% of the land being used for agriculture [14]. A parental questionnaire was used to identify 1458 children (aged 5 to 10 years) with different levels of farm and animal exposure; 1073 children (73.6% response rate) replied to an invitation to participate in this phase of the study, which included dust sampling at home. The study adhered to the tenets of the Declaration of Helsinki, and was carried out with ethical approval obtained from the Shropshire Research Ethics Committee, UK. Permission to visit the schools was obtained from the Senior Primary Advisor for Shropshire County Council [13]. Written consent from the parent / guardian of the participating child was obtained, in accordance with ethical approval [13].

### Questionnaires

The background questionnaire sent to all parents / guardians collected information on their child’s, sex, age, home environment, farming and animal exposure (whether this was current and/or in early life), diet, breast feeding history, health and atopic history (as well as family history) including wheezing, skin and nose symptoms. Responses were used to diagnose rhinitis and rhinoconjunctivitis using ISAAC definitions [8,13]. A separate eye questionnaire specifically inquired about their child’s ocular symptoms. This used as a basis the ISAAC rhino-conjunctivitis questions but modified these to allow all respondents to report conjunctivitis symptoms independent of the response to the question on watery-itchy eyes. The eye questionnaire also asked parents about specific environmental factors which may have caused the child’s conjunctivitis symptoms including: dust; flowers, grass or trees; contact with animals; and farming sprays (such as insecticides and pesticides).

### Clinical Survey

A single research team, including a paediatrician (MRP) and research nurse, carried out clinical assessments. Skin prick testing was undertaken on the volar surface of one forearm with the following allergens (ALK-Abelló, Horsholm, Denmark): dog hair, cat hair, horse hair, cow hair, 6-grass mix, house dust mite (Dermatophagoides pteronyssinus) and the following storage mites; (i) Acarus siro, (ii) Lepidoglyphus destructor, (iii) Tyrophagus putrescentiae. Any sized skin wheal was recorded as indicating IgE-mediated sensitization in accordance with the WAO guidelines [6].

### Definitions of Conjunctivitis, Rhinitis and Rhinoconjunctivitis

The presence of conjunctivitis was determined based on an affirmative response in the eye questionnaire to reporting itchy watery eyes when the child did not have a cold or the flu. Rhinitis was determined by an affirmative response to the ISAAC question in the background questionnaire: “has your child ever had a problem with sneezing, or a runny, or blocked nose when he/she did not have a cold or the flu?”.

Rhinoconjunctivitis could be determined in two ways: (1) respondents with conjunctivitis in the eye questionnaire were asked if the conjunctivitis was accompanied by rhinitis (using the ISAAC question above); (2) respondents with rhinitis in the background questionnaire were asked if the rhinitis was accompanied by conjunctivitis (using the ISAAC questions). The interrelationship between children with rhinoconjunctitivis identified by these two routes was also explored.

This analysis focussed on current reporting of these conditions (symptoms during the last 12 months). Seasonal symptoms were determined by the presence of symptoms exclusively in the six months from April to September. Children with symptoms in other months or in both parts of the year were designated as having perennial symptoms.

Evidence for an allergic basis for the symptoms was explored in two ways. Firstly amongst those ever reporting conjunctivitis symptoms (alone or with accompanying rhinitis) the association with parent reported specific environmental triggers (flowers, grass or tree pollen and house dust mite) was determined. Secondly, objective markers of sensitisation (positive skin prick test responses to house dust mite, pollen and animal danders) were investigated.

### Risk Factors in the Rural Environment

An analysis was then undertaken to determine the risk factors for these conditions with particular reference to the rural/farming environment.

### Statistical Analysis

Statistical analyses were carried out using STATA/SE software (Stata/SE 10 for Windows, StataCorp LP, College Station, TX, USA). Binomial confidence intervals on proportions with conjunctivitis, rhinitis and rhinoconjunctivitis were calculated. Odds ratios of the conditions by exposure were examined using logistic regression with and without adjustment for age, sex, breastfeeding status, family history of allergy, number of older and younger siblings. Adjustments were decided upon a priori.

## Results

Of the 1073 families who replied to invitation to take part, 919 completed the eye questionnaire (85.6%), of whom 894 children (49.9% male) had data on seasonality for both nasal and ocular symptoms. Of these children 768 underwent skin prick testing. Mean age of participants was 8.6 years (SD 1.8).

### Total Number Reporting Current Rhinitis Symptoms, Current Conjunctivitis Symptoms and Combined Rhinoconjunctivitis Symptoms (Table 1)

More children had parent-reported conjunctivitis than rhinitis. The majority of children with either rhinitis or conjunctivitis were affected by the other condition also: 64.1% (100/156) of those reporting conjunctivitis also reported rhinitis; 51.1% (69/135) of those reporting rhinitis also reported conjunctivitis. Children reporting both rhinitis and conjunctivitis symptoms in the same month at any point in the year resulted in a prevalence of 13.0% of children with parent reported rhinoconjunctivitis.

**Table 1:** Prevalence of allergic conjunctivitis, rhinitis and rhinoconjunctivitis: seasonality, environmental triggers and sensitization.

	None	Conjunctivitis		Rhinitis		Rhinoconjunctivitis	
No. of children (N = 894)	676	156		135		116	
Prevalence (95% CI)	-	17.5%		15.1%		13.0%	
		(15.0, 19.9%)		(12.7, 17.5%)		(10.8, 15.2%)	
Seasonality	-	Seasonal	Perennial	Seasonal	Perennial	Seasonal	Perennial
		101 (64.7%)	55 (35.3%)	63 (46.7%)	72 (53.3%)	107 (92.2%)	9 (7.8%)
Environmental triggers:*							
*Flowers*, *grass or tree pollen*	1.2%	84.2%	34.6%	-	-	85.1%	44.4%
*House dust mite*	0.3%	10.9%	29.1%	-	-	14.0%	55.6%
*Animals*	0.4%	27.7%	36.4%	-	-	29.9%	44.4%
Sensitization:							
*Grass pollen*	5.2%	59.1%	42.6%	57.1%	30.8%	61.7%	37.5%
*House dust mite*	7.1%	29.6%	42.6%	30.4%	30.8%	33.0%	50.0%
*Animals*	4.8%	39.8%	40.4%	37.5%	33.9%	41.5%	50.0%

*Triggers identified as ever having caused conjunctivitis symptoms

In this survey, 63.6% of the children with parent reported hay fever had had this diagnosis confirmed by a doctor. In contrast, only 5.8% of the children with itchy watery eye symptoms had had a diagnosis of allergic eye disease made by a doctor. This compares with asthma where 99.1% of the children with parent reported asthma had had the diagnosis confirmed by a doctor and eczema where the figure was 88.3%.

Markedly different seasonal patterns were observed for those with conjunctivitis, rhinitis or rhinoconjunctivitis. Two thirds (64.7%) of children with conjunctivitis symptoms had seasonal conjunctivitis, half with rhinitis symptoms (46.7%) had seasonal rhinitis and over nine out of ten (92.2%) with rhinoconjunctivitis symptoms had seasonal rhinoconjunctivitis. The end result of this being that amongst children with seasonal symptoms, conjunctivitis was more likely to predominate over rhinitis symptoms, whereas the reverse occurred amongst those with perennial symptoms.

The environmental triggers of ocular symptoms reflected the seasonal demarcation. Those with seasonal conjunctivitis or rhinoconjunctivitis predominantly identified pollens as a trigger, although over a quarter reported animal triggers as well. House dust mite as a trigger was reported by one in ten children with seasonal conjunctivitis or rhinoconjunctivitis symptoms. In contrast those children with perennial eye symptoms were much less likely to report pollens and more likely to report house dust mite and animal danders than seasonal sufferers.

Sensitization on skin prick testing revealed a more attenuated difference in sensitisation between seasonal and perennial children compared with the parent identified triggers. There was also an interesting pattern of discordance between environmental triggers and sensitization depending upon the seasonality of symptoms. Amongst those with seasonal symptoms, sensitisation to grass pollen was significantly less than parent recognition of it as a trigger. Conversely, house dust mite and animal dander sensitisation was significantly more common than these allergens being recognised as triggers. Amongst those with perennial symptoms the trend for grass pollen was the reverse, with more children being sensitised than had a parent identified pollen trigger. For house dust mite and animals sensitisation exceeded recognition of them as a trigger, but not to the same extent as for those with seasonal symptoms.

### Monthly Reporting of Rhinitis, Conjunctivitis and Rhinoconjunctivitis (Fig 1)

**Fig 1:**
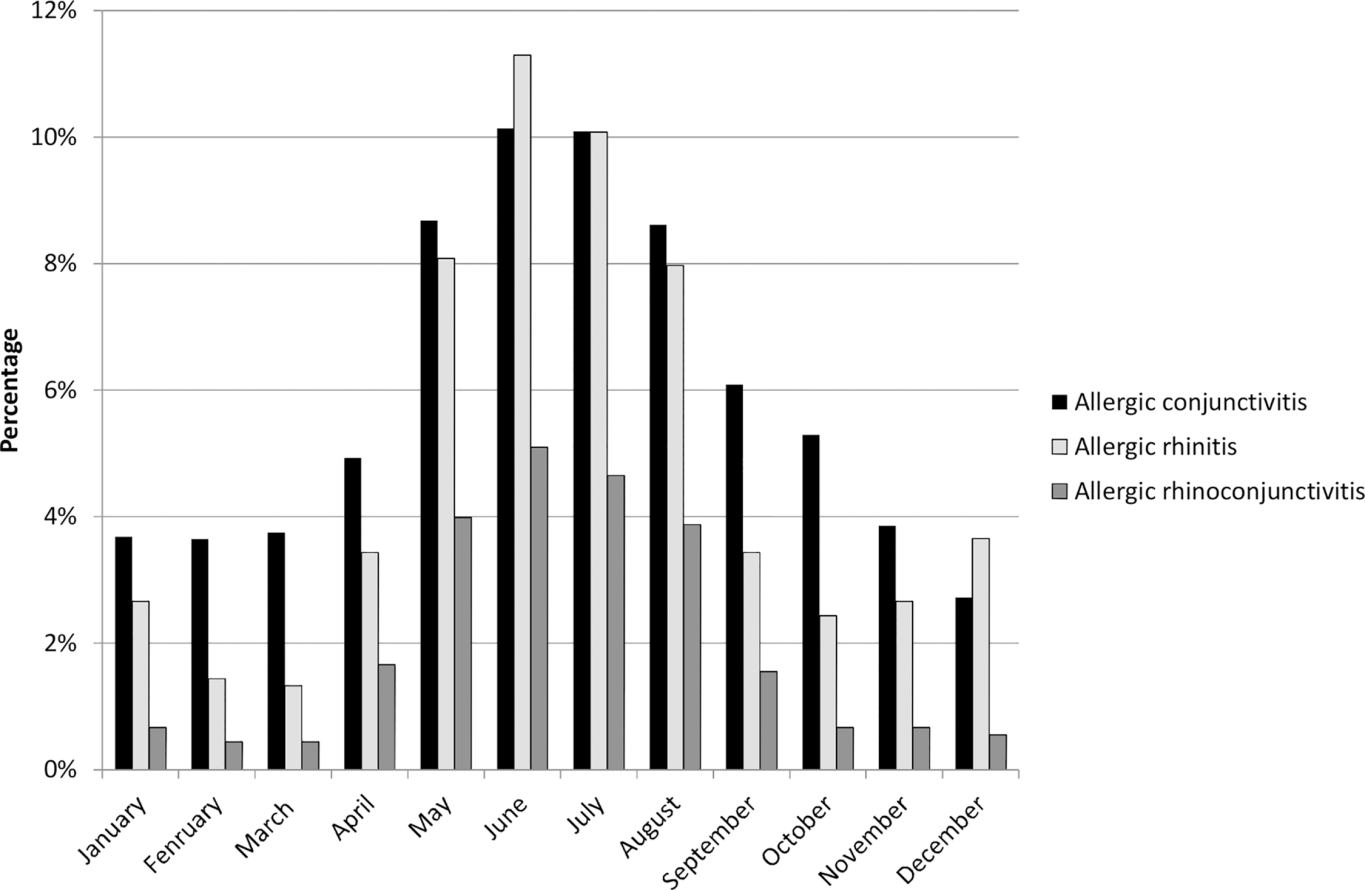
Seasonal variation in Allergic Rhinitis/Conjunctivitis/Rhinoconjunctivitis current symptoms

Exploring this seasonal pattern further, Fig 1 shows the month by month prevalence of symptoms for the three conditions. Whilst conjunctivitis and rhinoconjunctivitis show a clear seasonal trend with a summer peak and winter waning, rhinitis in contrast shows a clear biphasic distribution with a summer peak but a lesser winter peak with February/March and October being the months with lowest prevalence. This biphasic distribution is not explained by any difference in house dust mite sensitisation rates and suggests that parents ability to differentiate rhinitis due to colds or flu in winter is perhaps somewhat limited.

### Inter-Relationship between Reporting on Rhinitis and Conjunctivitis Symptoms (S1 Fig)

The background questionnaire identified 78 participants with rhinitis who also had itchy watery eyes. However the problem of using a nested question to determine the prevalence of conjunctivitis was demonstrated with the eye questionnaire identifying a further 99 children with conjunctivitis symptoms, not picked up by the standard rhinitis ISAAC question. Half of these had rhinitis symptoms accompanying their eye symptoms.

There were a number of seemingly discordant subgroups identified by this intermeshing of the two questionnaires. An obvious example being the 44 children with conjunctivitis associated with rhinitis who reported no rhinitis with or without conjunctivitis in the background questionnaire. Similarly 18 children with rhinitis on the background questionnaire accompanied by itchy watery eyes denied any symptoms of itchy watery eyes in the eye questionnaire.

The implication of these results would be that a significant proportion of parents perceive their children presenting with primarily rhinitis or primarily conjunctivitis as two separate conditions. Hence a parent can have a child who gets episodes of conjunctivitis that have been accompanied by rhinitis but denies having a child who gets episodes of rhinitis that have been accompanied by conjunctivitis.

### Inter-Relationship between Parent Reported Symptoms, Triggers and Sensitisation (S2 to S4 Figs)

The issue of the discrepancy observed in Table 1 between parents recognising a trigger for their child’s conjunctivitis symptoms and sensitisation was explored further for pollen (S2 Fig), house dust mite (S3 Fig) and animal danders (S4 Fig).

Of the 106 children who had recognised pollen as ever having triggered eye symptoms and the 116 children who were grass pollen sensitised on skin prick testing, only 60 children were both (52% of those grass pollen sensitised and 57% of those recognising pollen as a trigger). Asymptomatic sensitisation was common: 44 (38%) sensitised children had no current SAC or SAR. Of the 25 children with pollen ever having caused eye symptoms, but with no current SAC or SAR, only 8 (32%) were grass pollen sensitised. This compares with the 75 children with current SAC and pollen as a recognised trigger of whom 48 (64%) were grass pollen sensitised. This suggests that in the absence of current symptoms (SAC or SAR) a parent suspicion of a pollen trigger is a poor predictor of sensitisation with two thirds of such children showing no evidence of grass pollen sensitisation.

There were less children with perennial conjunctivitis symptoms but amongst these a different pattern was seen. Of the 27 children with house dust mite recognised as a trigger of AC symptoms and the 104 house dust mite sensitised children, 17 were both—63% of those recognising house dust mite as a trigger (similar to grass pollen) but only 16% of those who were house dust mite sensitised. This reflects the fact that asymptomatic sensitisation to house dust mite was very common– 64 children (61.5% of house dust mite sensitised children) had no current AC or AR. The same pattern was seen with regards to parent suspected triggers and sensitisation. Four of the nine children (44%) without any AC or AR symptoms but whose parents suspected house dust mite had ever been a trigger of AC symptoms were sensitised. This compares with 9 of the 13 children (69%) with current AC symptoms and a house dust mite suspected trigger being sensitised.

Of the 50 children with animals suspected of being a trigger and the 92 who were sensitised to an animal dander, 36 were both. This represents 72% of those recognising animals as a trigger (the highest for all three external triggers) and 39% of those animal sensitised. Asymptomatic animal sensitisation was again common with 45 children (49%) of those sensitised having no AC or AR. Parent suspicion of animals ever having caused conjuncitivitis symptoms in the absence of current symptoms was more likely to be predictive of sensitisation than for grass pollen and house dust with 14 out of 24 being sensitised (58%), but this compares with a sensitisation rate of 89% (16/18) amongst those with current eye symptoms.

Ocular symptoms significantly exceeded nasal symptoms for those with grass pollen sensitisation: 49% (57/116) versus 30% (35/116). However for the perennial triggers ocular and nasal symptoms were similar for those with house dust mite sensitisation (19% versus 22%) and animal sensitisation (21% versus 24%).

### Risk Factors for Rhinitis, Conjunctivitis, and Rhinoconjunctivitis in a Rural Population (Table 2)

As one would anticipate both family and child history of allergy were strongly related to an increased risk of rhinitis (R), conjunctivitis (C), and rhinoconjunctivitis (RC), especially a personal history of atopic disease (eczema and particularly asthma) (Table 2). Having older siblings reduced the risk of RC (Table 2). Family size was strongly and statistically significantly related to both C and RC, with a trend for the latter with increasing family size.

**Table 2:** Atopic, familial and environmental associations with allergic conjunctivitis, rhinitis and rhinoconjunctivitis symptoms.

	Prevalence of exposure	Conjunctivitis		Rhinitis		Rhinoconjunctivitis	
	n (%)	OR^adj^ *(95%CI) p value		OR^adj^ *(95%CI) p value		OR^adj^ *(95%CI) p value	
**Atopic history**							
Any family history of allergies	689 (77.1)	2.72 (1.53–4.82)	0.001	2.74 (1.46–5.16)	0.002	4.49 (2.03–9.95)	<0.0005
Asthma (any relative)	419 (46.9)	1.75 (1.20–2.54)	0.004	1.62 (1.08–2.43)	0.02	1.95 (1.27–3.00)	0.002
Hay fever (any relative)	448 (50.1)	2.48 (1.67–3.68)	<0.0005	2.38 (1.55–3.66)	<0.0005	3.62 (2.23–5.87)	<0.0005
Eczema (any relative)	454 (50.8)	2.08 (1.41–3.06)	<0.005	2.41 (1.57–3.69)	<0.0005	2.52 (1.60–3.96)	<0.0005
Current child eczema	113 (12.7)	2.74 (1.71–4.11)	<0.0005	3.22 (1.97–5.25)	<0.0005	2.83 (1.69–4.75)	<0.0005
Current child asthma	122 (13.8)	5.91 (3.74–9.32)	<0.0005	7.20 (4.49–11.5)	<0.0005	6.93 (4.25–11.3)	<0.0005
**Family circumstances**							
Any older siblings	500 (56.1)	0.78 (0.54–1.22)	0.18	0.87 (0.58–1.30)	0.50	0.65 (0.43–0.99)	0.04
Any younger siblings	497 (55.8)	1.01 (0.69–1.46)	0.97	0.82 (0.55–1.23)	0.34	0.81 (0.53–1.23)	0.32
Family size: 1	59 (6.7)	1.0		1.0		1.0	
2	441 (50.2)	0.35 (0.18–0.67)	0.002	0.58 (0.28–1.20)	0.14	0.37 (0.18–0.75)	0.006
3	247 (28.1)	0.43 (0.21–0.86)	0.02	0.62 (0.29–1.35)	0.23	0.35 (0.16–0.75)	0.007
4+	132 (15.0)	0.36 (0.16–0.78)	0.01	0.41 (0.17–1.00)	0.05	0.26 (0.11–0.62)	0.003
**Farming variables**							
Non farmers	478 (53.5)	1.0		1.0		1.0	
Parent(s) work on a farm	127 (14.2)	0.69 (0.39–1.23)	0.21	0.99 (0.54–1.82)	0.98	0.82 (0.42–1.57)	0.54
Live and work on a farm	289 (32.3)	0.47 (0.29–0.79)	0.004	0.57 (0.33–1.01)	0.05	0.57 (0.32–1.03)	0.06
Current horse / pony exposure	300 (33.6)	0.65 (0.43–0.99)	0.05	0.84 (0.54–1.31)	0.44	0.56 (0.34–0.92)	0.02
Current farm animal exposure	442 (49.5)	0.71 (0.48–1.04)	0.08	0.63 (0.41–0.97)	0.04	0.80 (0.51–1.25)	0.32
Early farm animal exposure	321 (39.4)	0.52 (0.33–0.80)	0.003	0.53 (0.33–0.85)	0.009	0.56 (0.34–0.92)	0.02
Child drinks unpasteurised milk	207 (23.8)	0.46 (0.27–0.77)	0.003	0.23 (0.11–0.48)	<0.0005	0.50 (0.28–0.90)	0.02
Child plays in a barn	663 (74.9)	0.61 (0.39–0.95)	0.03	0.64 (0.42–0.98)	0.04	0.65 (0.40–1.04)	0.07

*Odds ratios (OR) adjusted for age, sex, ever breast fed, month of examination, family history of allergy (except atopic history variables), number of older and younger siblings (except family size /order variables)

There were no associations with other variables including diet, use of household fuels and breastfeeding (data not presented). Despite this breastfeeding status was adjusted for in the multivariable analysis, as this is often regarded as a potential confounder.

### Rural Risk Factors for Rhinitis, Conjunctivitis, and Rhinoconjunctivitis

Children were classified into one of three exposure groups; farming children whose parents live and work on a farm (n = 291), labourer’s children whose parent(s) work on a farm but do not live on a farm (n = 130), and control children (n = 496). For all three conditions, control children had the highest prevalence, labourer’s children intermediate levels and farm children the lowest levels with the protective effect being statistically significant for AC and borderline for the other two conditions.

**Fig 2:**
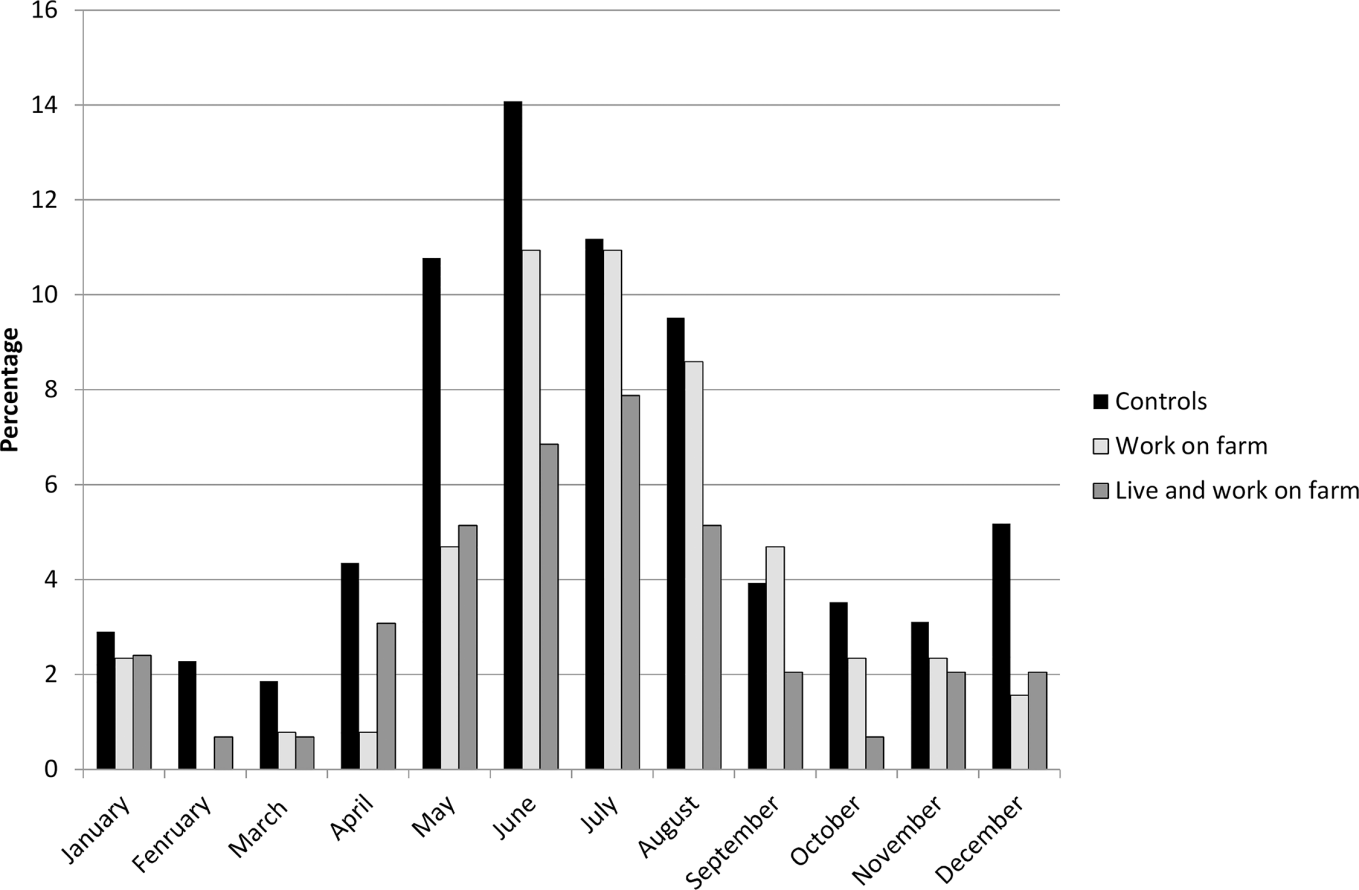
Seasonal variation in conjunctivitis symptoms by farming status.

We investigated whether there was a seasonal pattern to this protective effect with particular reference to AC. Monthly reporting of AC symptoms by farm exposure group is shown in Fig 2. In general there was a marked stepwise reduction in AC symptoms with control children having the highest prevalence, farm labourer’s children having intermediate levels and farm children the lowest. The reduction showed no seasonal variation being present in winter months as much as summer months.

All farming related variables were associated with a lower risk of all three conditions (i.e. odds ratios less than 1), with the strongest statistically significant effects in the adjusted analyses seen for early farm animal exposure (in the first year of life), consumption of unpasteurised milk and playing in a barn or stable.

## Discussion

Whilst the WAO suggested that AC should be seen as a companion symptom to AR, our study showed that the prevalence of AC exceeded AR. Furthermore it seemed clear that families perceived the two conditions as separate entities with the other condition sometimes occurring in conjunction with the primary condition. Just as has been observed in Oxfordshire, remarkable was the infrequency with which children suffering from eye symptoms end up with a doctor confirming a diagnosis of allergic eye disease, in contrast to hay fever, asthma or eczema. We did not expect to see such a marked seasonal variation between the three conditions, with ARC occurring almost exclusively in summer, with rhinitis being split fifty-fifty, and seasonal conjunctivitis prevailing in two thirds of AC children. All three conditions were at their peak incidence in June and July, consistent with the NHANES III study where ocular symptoms peaked in the same months [7].

The difficulty of relying on parent identification of external triggers for symptoms as a marker of sensitisation was demonstrated, particularly in the absence of current symptoms. Equally significant was the poor predictive value of sensitisation as a predictor of an external trigger being recognised by families or, for that matter, it causing any symptoms.

Ocular symptoms predominated amongst those grass pollen sensitised whereas the split between ocular and nasal symptoms was even for house dust mite and animal sensitisation. This contrasts with the NHANES III results were ocular symptoms were more frequent in relation to animals, house dust mite and pollen [7].

Despite the difference in seasonality and patterns of reported triggers between the three conditions this appeared to have no bearing on the protective effect observed amongst farming children in this rural population. The protective effect was apparent all year around and not influenced by seasonality. Specific factors within the farming environment that conferred a protective effect were similar to those observed in the previous farming literature for asthma and sensitisation–early farm animal exposure [9,15–17], playing in barns and stables and consumption of unpasteurised milk [13]. This protective effect appears to be sustained [18]. The graded protective effect of increasing numbers of older siblings on SAC, is also akin to findings for other allergic outcomes [19].

Although we were able to show a protective association between farming and these conditions, and that exposure to farm animals appeared to be important, we did not have the power to dissect further which part of the farming environment might be responsible. For instance, we were unable to differentiate between type of farming as a high proportion (40%) of farms were mixed arable and livestock with 50% pure livestock and only 7% pure arable. However, early farm animal exposure was more protective than current exposure, which has been suggested as being important in other studies [17].

This study achieved a good response rate given that the first phase of the study was carried out during the worst flooding in Shropshire since the late 1940’s, and the final phase was during the last UK foot and mouth outbreak [20], which restricted access to many rural areas in Shropshire.

Limitations of the study include the potential for participation bias with families with a history of atopic disorders being more likely to participate in a study about asthma and allergy. The study was also cross sectional so it is difficult to know whether families with allergic histories / symptoms avoid farming related occupations. However migration out of farming was asked about in children participating in the larger study and did not account for the associations observed. If such an effect were to exist, it is unlikely to fully account for the consistent associations observed between farming status and a raft of allergic diagnoses. Only longitudinal studies will be able to formally establish the presence of any potential selection effects.

## Conclusions

Allergic disorders are a huge public health burden and current research into allergens will be an important component of managing this condition in the future, both in terms of prevention and treatment. Whilst disease modifying (and potentially curing) treatment for SAC is available with immunotherapy, this is expensive and not hazard free. Factors are present in the farming environment that have potent immunological effects. Stable dust has been shown to have a broad immunosuppressive effect, perhaps explaining why a broad suppression of the different conditions was observed. Establishing the exact constituents of the farm environment, whether it be in dust or unpasteurised milk remains to be achieved. However the protective effects observed in this and other studies from the farming environment make this an important task to pursue.

## Supporting Information

S1 Data PLoS One_Shropshire RC_Data.csv. (CSV)

S1 Fig Interrelationship between current rhinitis and conjunctivitis symptoms. (TIF)

S2 Fig Interrelationship between seasonal rhinitis, seasonal conjunctivitis, pollen as a trigger of symptoms and grass pollen sensitisation. (TIF)

S3 Fig Interrelationship between perennial rhinitis, perennial conjunctivitis, dust as a trigger of symptoms and house dust mite sensitisation. (TIF)

S4 Fig Interrelationship between perennial rhinitis, perennial conjunctivitis, animals as a trigger of symptoms and animal sensitisation. (TIF)

## Abbreviations

WAO: World Allergy Organization
AC: Allergic Conjunctivitis
SAC: Seasonal Allergic Conjunctivitis
AR: Allergic Rhinitis
SAR: Seasonal Allergic Rhinitis
ARC: Allergic Rhinoconjunctivitis
