# Supplementary figures and images for: Inter-Relationship between Rhinitis and Conjunctivitis in Allergic Rhinoconjunctivitis and Associated Risk Factors in Rural UK Children
Source: PLoS One. 2015 Nov 24;10(11):e0143651. doi: 10.1371/journal.pone.0143651 (PMC4658044; doi:10.1371/journal.pone.0143651)

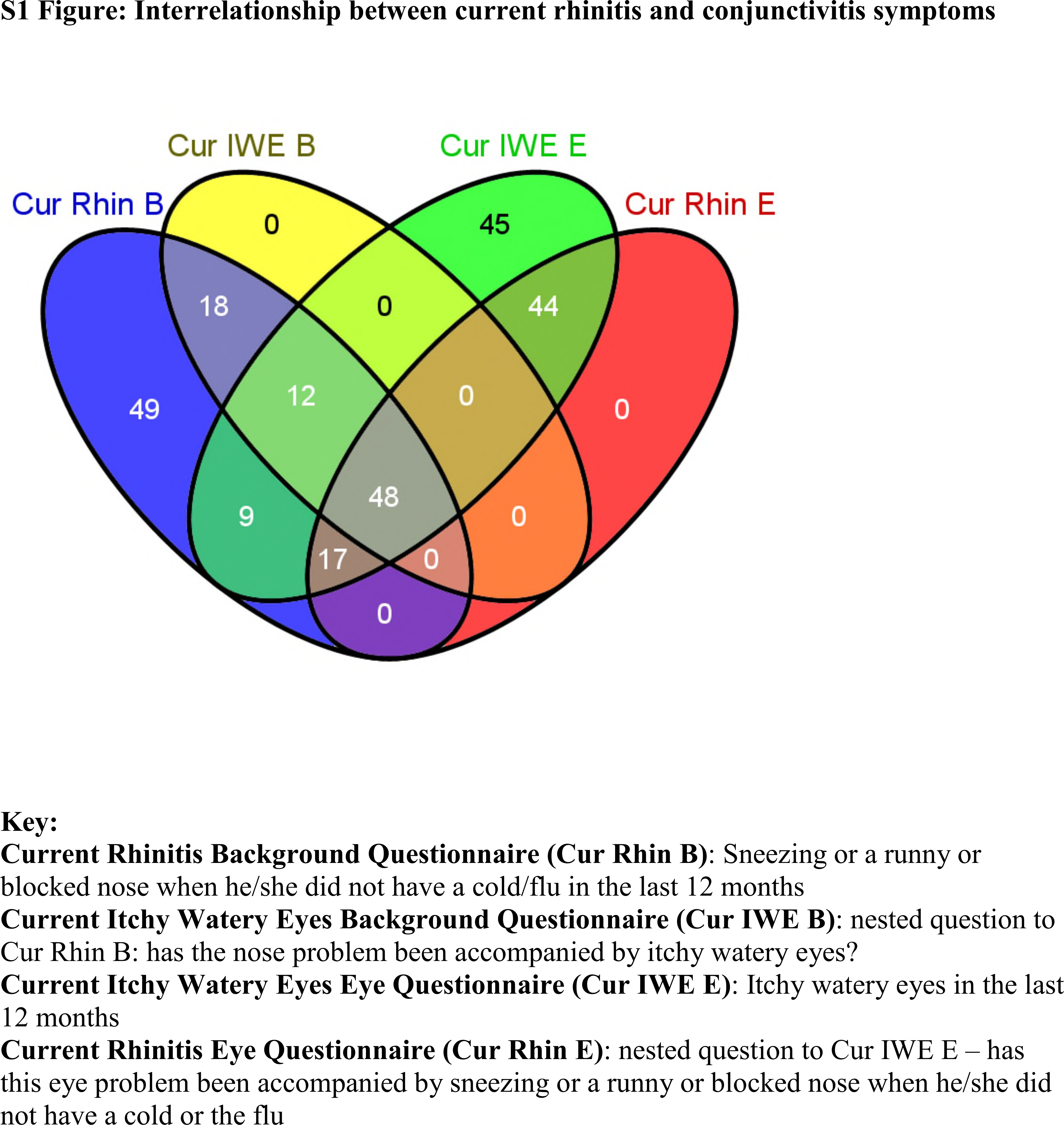

Supplement: S1 Fig — (TIF) [file pone.0143651.s002.tif]

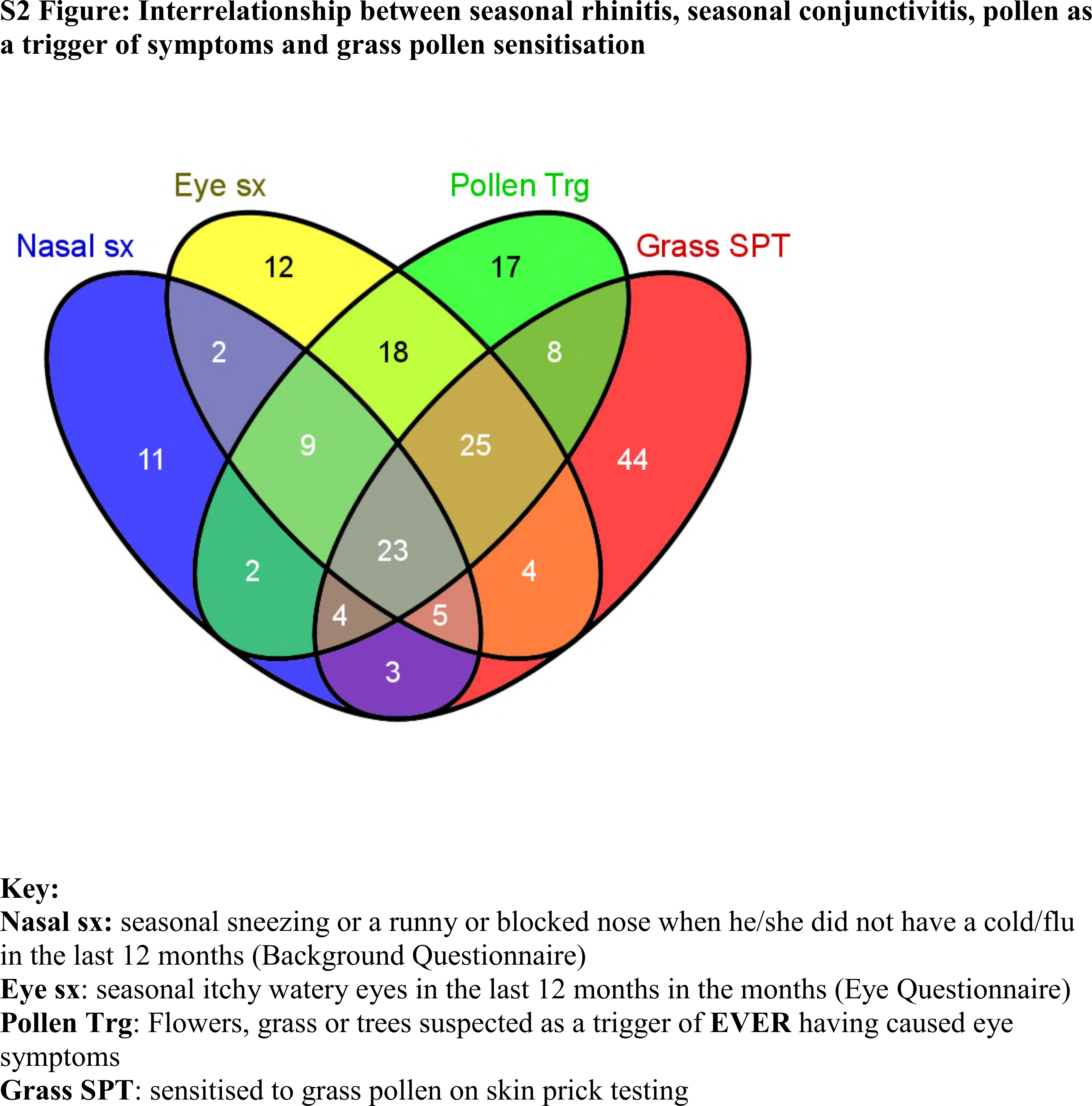

Supplement: S2 Fig — (TIF) [file pone.0143651.s003.tif]

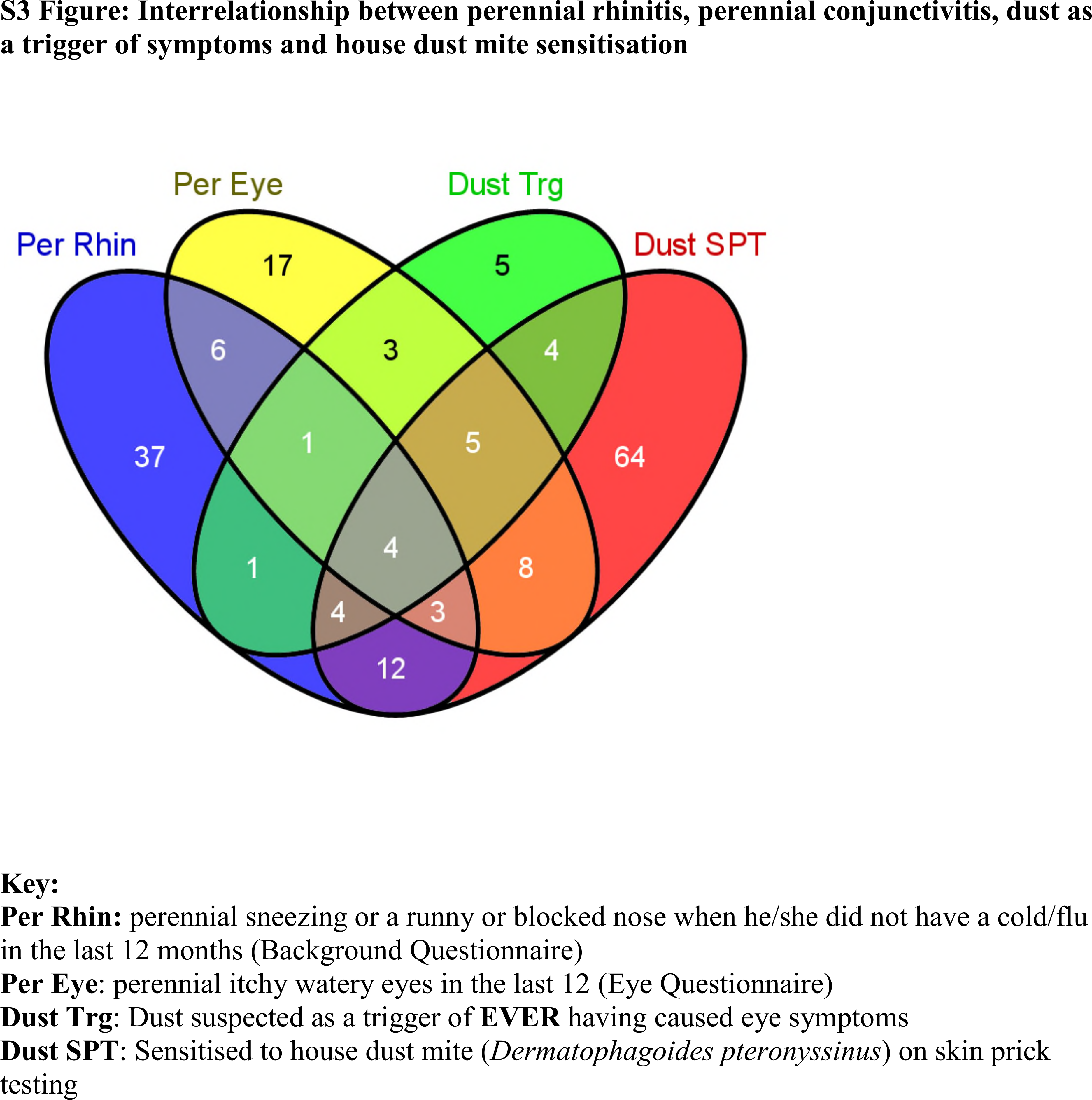

Supplement: S3 Fig — (TIF) [file pone.0143651.s004.tif]

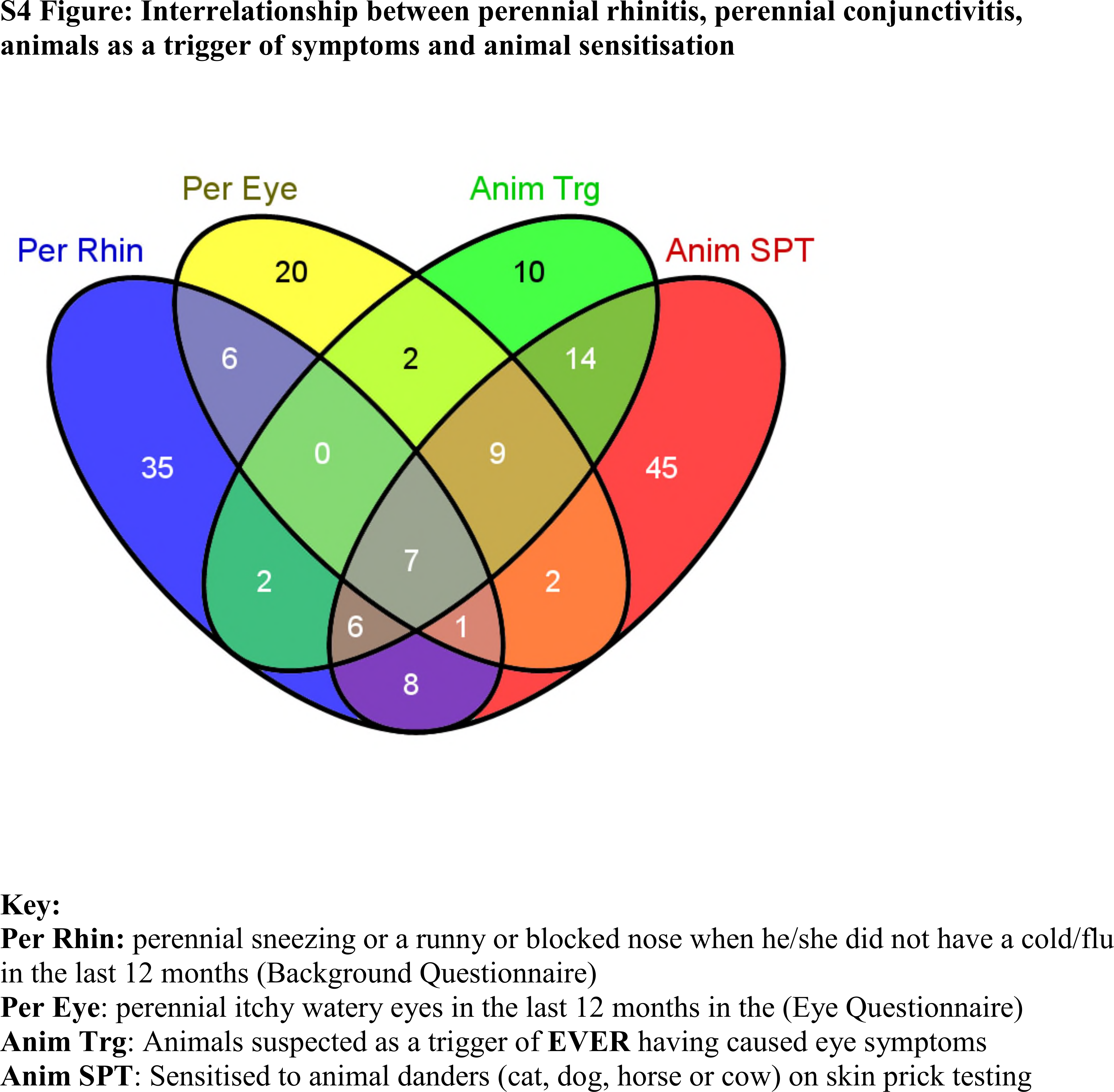

Supplement: S4 Fig — (TIF) [file pone.0143651.s005.tif]
